# Temperature-dependent competitive advantages of an allelopathic alga over non-allelopathic alga are altered by pollutants and initial algal abundance levels

**DOI:** 10.1038/s41598-020-61438-9

**Published:** 2020-03-10

**Authors:** Yongeun Kim, Jino Son, Yun-Sik Lee, June Wee, Minyoung Lee, Kijong Cho

**Affiliations:** 1 Ojeong Resilience Institute, Korea University, Seoul, 02841 Republic of Korea; 2 Department of Environmental Science and Ecological Engineering, Korea University, Seoul, 02841 Republic of Korea

**Keywords:** Ecological modelling, Freshwater ecology, Microbial ecology, Population dynamics

## Abstract

In the context of climate warming, the dominance of allelopathic algae that cause ecosystem disturbances is an important topic. Although the hypothesis that an increase in temperature will be favorable to the dominance of allelopathic algae has been increasingly supported by many studies, it is still unclear how other factors can affect the influence of temperature. In this study, the effects of copper exposure and initial algal abundance on the competition between *Pseudokirchneriella subcapitata* (non-allelopathic alga) and *Chlorella vulgaris* (allelopathic alga) were investigated during temperature changes. The results showed that increased temperatures enhanced the competitive advantage of *C. vulgaris* only in the absence of copper exposure. Our data confirmed that copper exposure along with increased temperature (20–30 °C) may change the competitive advantage of *C. vulgaris* from favorable to unfavorable. The initial algal abundance was found to affect competition outcome by controlling copper toxicity. This study suggests that pollutants and initial abundance can alter the effects of increased temperature on the allelopathic interaction. Given the temporal dynamics of algal abundance and the pollutants in natural ecosystems, these findings should be considered in the prediction of temperature influence on an algal community.

## Introduction

Competition among algal species is an important ecological process that determines the structure and functions of the algal community in aquatic ecosystems^1^. Algae not only undergo growth competition at limited resources but also interact with each other through interference competition, which affects the growth of competitors. These competitions form an ecological mechanism that allows various algal species to coexist, but it is also a process in which certain algal species dominate the habitat under specific environmental conditions^2^. The dominance of certain algal species through the competition often breaks the balance of the species in aquatic environments and inhibits the ecological function of the community^3^. Particularly, some allelopathic algae, which affect the growth and development of their competitors by producing secondary metabolites called allelochemicals^4^, grow rapidly causing algal blooms, which is becoming an urgent global issue owing to the serious economic damage that they cause and disturbances of aquatic ecosystems worldwide^5^. Given that most algal blooms are caused by the dominance of a single algal species^3^, an understanding of the competition among algal species is becoming increasingly important for effective algal bloom management.

Predicting the influence of climate warming on the algal community is a critical priority because of the increasing evidence of temperature-induced direct and indirect effects on many ecosystems^6,7^. It is obvious that the structure and function of algal communities change owing to climate warming because temperature is an influential environmental driver of the biological processes that determine the growth rates, nutrient stoichiometry, and spatial distribution of algae in aquatic ecosystems^1,8^. Some studies suggest that an increase in temperature may make allelopathic algal species more competitive in aquatic ecosystems^5,6^. Jöhnk *et al*.^9^ and Granéli *et al*.^6^ have reported that higher temperatures (often above 25 °C) can accelerate the growth of allelopathic algae and, thus, promote the formation of harmful algal blooms. Furthermore, Trochine *et al*.^10^ and Ma *et al*.^5^ have shown that increased temperatures enhance the competitive advantage of allelochemical-producing species by increasing the allelopathic effect. These experimental findings have been fueling the hypothesis that increased temperature will enhance the competitive advantage of allelopathic algae over their competitors. Several well-designed long-term field studies have produced results supporting this hypothesis^11,12^.

Nonetheless, it is still uncertain how the algal community in actual ecosystems is affected by increased temperature because the interaction of increasing temperature with other environmental factors may yield complex and unpredictable effects^7^. For example, nutrient conditions^13^, CO_2_ levels^7,14^, photoperiods^15^, and water pH^16^ may alter the effects of the increase in temperature on the ecological interaction among algal species. Aside from these physicochemical factors, environmental pollutants are likely to be some of the key factors that affect the algal competition in actual ecosystems under warm conditions. The pollutants not only are a substantive factor that frequently flows into aquatic ecosystems either naturally or anthropogenically^17^ but also can exert severe effects at the community level beyond the individual level^18^. Besides, changes (as a result of pollutants) in the susceptibility of organisms to environmental factors allow pollutants and other environmental stressors to often have stronger-than-additive effects^19^. Therefore, the effect of temperature changes on the competition between allelopathic and non-allelopathic algae is expected to be altered by pollutant exposure. Several research groups have experimentally demonstrated that pollutants can change the outcome of algal competition^21,22^, but the effect of both pollutants and temperature changes have not been considered in these studies. The hypothesis that increased temperature enhances the competitive advantage of allelopathic algal species needs to be tested under pollutant exposure.

In this study, combined effects of a pollutant and initial abundance of algae on the competitive advantage of an allelopathic alga were examined under changing temperature conditions. Copper was chosen as the environmental pollutant, because it is a trace metal highly toxic to algae, despite being an essential element for metabolic processes in these life forms^20^. In addition, copper is one of the heavy metals frequently found in freshwater ecosystems^17^. Another factor for testing was initial algal abundance, which is closely related to the success of competitive dominance of allelopathic species^23^. The initial abundance may also serve as a factor leading to the preoccupancy effect of a particular species on algal succession or community assemblage; this phenomenon is called the priority effect^23,24^.

To investigate the influence of the pollutant and initial abundance together with temperature on algal competition, laboratory level experiments and simulations were conducted using a mathematical model. Two species of algae, *Pseudokirchneriella subcapitata* (formerly known as *Raphidocelis subcapitata* and *Selenastrum capricornutum*, a non-allelopathic alga) and *Chlorella vulgaris* (an allelopathic alga) were selected as the model species. These species are widely distributed in freshwater ecosystems and are well known for their allelopathic interactions^25,26^. Specifically, the growth of *P. subcapitata* and *C. vulgaris* in a coculture system in the presence of various combinations of the three factors was simulated by the model, which was calibrated using data obtained from a prolonged experiment (55 d). This approach helped us to investigate (1) the influence of temperature on the interference competition between *P. subcapitata* and *C. vulgaris*, (2) the effect of copper exposure levels and initial abundance levels on the interference competition, and (3) the effects of temperature on the interference competition at different copper exposure levels and initial abundance levels.

## Results

### Experimental results and model calibration

The experimental results clearly showed that the initial abundance levels of *P. subcapitata* and *C. vulgaris*, temperature, and copper exposure levels had an influence on the abundance dynamics of both algal species in coculture (Supplementary Figs. S1–S3). The increase in the initial abundance of each algal species exerted a positive effect on their growth in coculture, regardless of temperatures and copper concentrations. The growth and decline of both algal populations were promoted by increasing temperatures, except for the data point at 35 °C, where the growth of both algal species was very slow. Copper exposures had a direct effect on the competitive advantage of the two algal species. For example, *C. vulgaris* predominated in the absence of copper exposure, but the opposite was true for *P. subcapitata*. Detailed outcomes of each combination can be found in Supplementary Figs. S1–S3, and the effects of the factors on the interference competition are explored in more detail in the simulations in the next section.

The parameters were estimated using experimental data before the simulation, and the estimated values are listed in Supplementary Tables S1 and S2. The line predicted by the calibrated model manifested good agreement with the observed data for both *P. subcapitata* and *C. vulgaris* (Supplementary Figs. S1–S3). The performance of the model was evaluated using PBIAS (percent bias), the IoA (index of agreement), and ME (model efficiency), showing that the calibrated models adequately described the competitions in the simulation (Supplementary Table S3). All PBIAS values were within 15%; the evaluation standard of a satisfactory model has been suggested by Moriasi *et al*.^27^: ranging from 4.22% to 7.45% for *P. subcapitata* predictions and from 1.31% to 6.52% for *C. vulgaris* predictions. The IoA and ME estimates were greater than 0.9 and were acceptable (IoA>0.7, ME>0.7) according to Moriasi *et al*.^27^ and Saseendran *et al*.^28^.

### Competition of *P. subcapitata* and *C. vulgaris* at different temperatures and copper exposure levels

**Figure 1:**
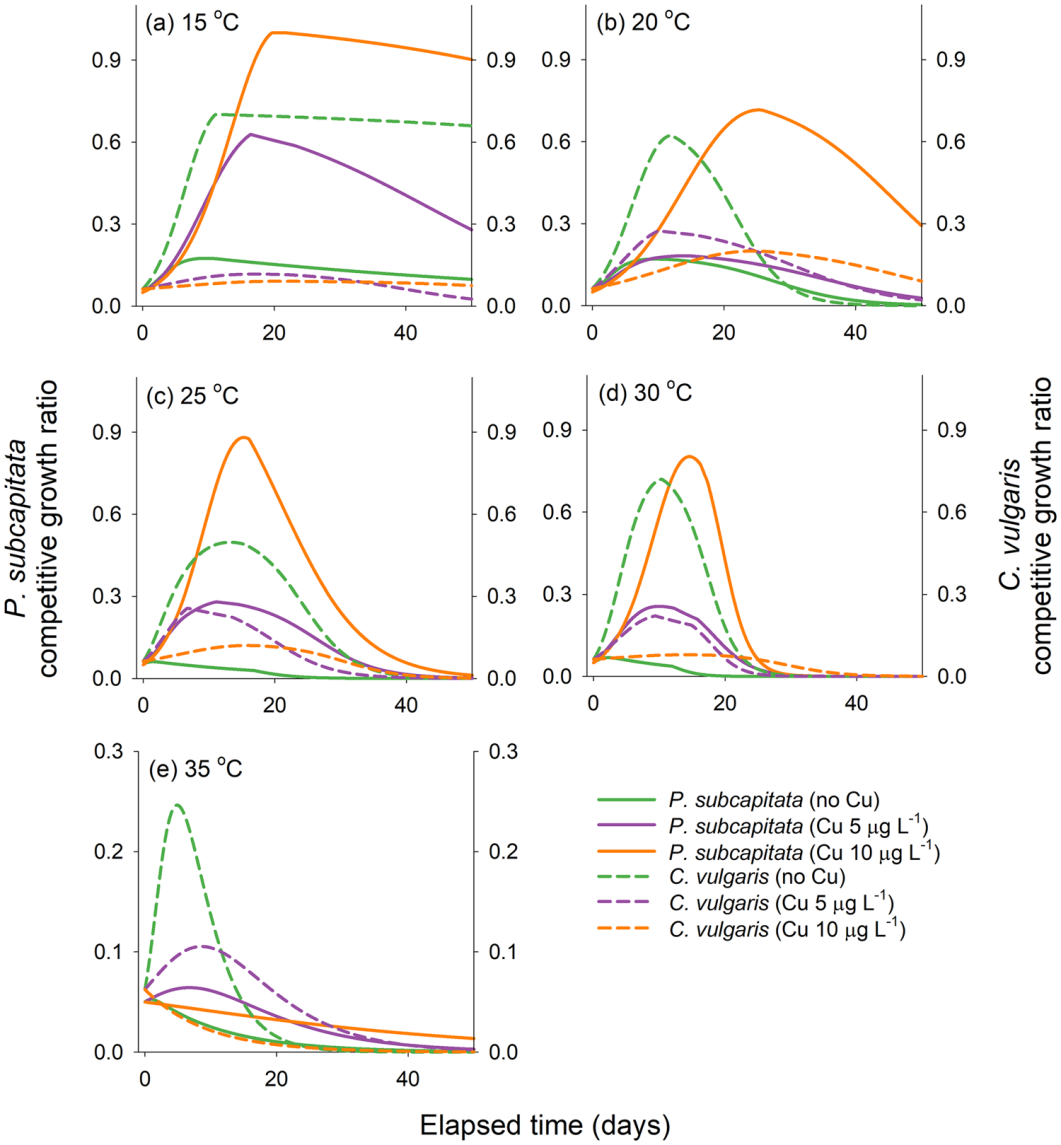
Line plots for the competitive growth ratio of *Pseudokirchneriella subcapitata* and *Chlorella vulgaris* in coculture at (**a**) 15 °C, (**b**) 20 °C, (**c**) 25 °C, (**d**) 30 °C, and (**e**) 35 °C at three copper exposure levels: 0, 5, and 10 μg L^−1^. The initial abundance of *P. subcapitata* was 2.5 × 10^5^ cells mL^−1^, and that of *C. vulgaris* was 2.5 × 10^6^ cells mL^−1^.

The simulation results revealed that the competitive growth ratio (i.e., the ratio of maximum algal abundance in coculture to that in single culture) of *P. subcapitata* and *C. vulgaris* was affected by both temperature and copper concentration (Fig. 1). It was found that a temperature increase from 15 to 30 °C promoted the growth and decline of the two competing populations, and copper changed the dominant species in coculture. In particular, the competitive growth ratio of *C. vulgaris* in the absence of copper exposure reached 0.50 at all temperatures other than 35 °C and that of *P. subcapitata* was below 0.17 under all the temperature regimes. Although the competitive advantage of the allelopathic alga *C. vulgaris* was evident in the absence of copper exposure, *P. subcapitata* became dominant over *C. vulgaris* in interference competition as copper exposure levels increased. For instance, when the copper concentration increased from 5 to 10 μg L^−1^ at 20 °C, the competitive growth ratios of *P. subcapitata* and *C. vulgaris* changed from 0.19 and 0.27 to 0.73 and 0.19, respectively (Fig. 1b). The simulation results also indicated that the growth of the two algal species significantly diminished at 35 °C, and in particular, it was found that both species could not grow at 10 μg L^−1^ copper.

### Influence of initial abundance of a competitor at different temperatures

**Figure 2:**
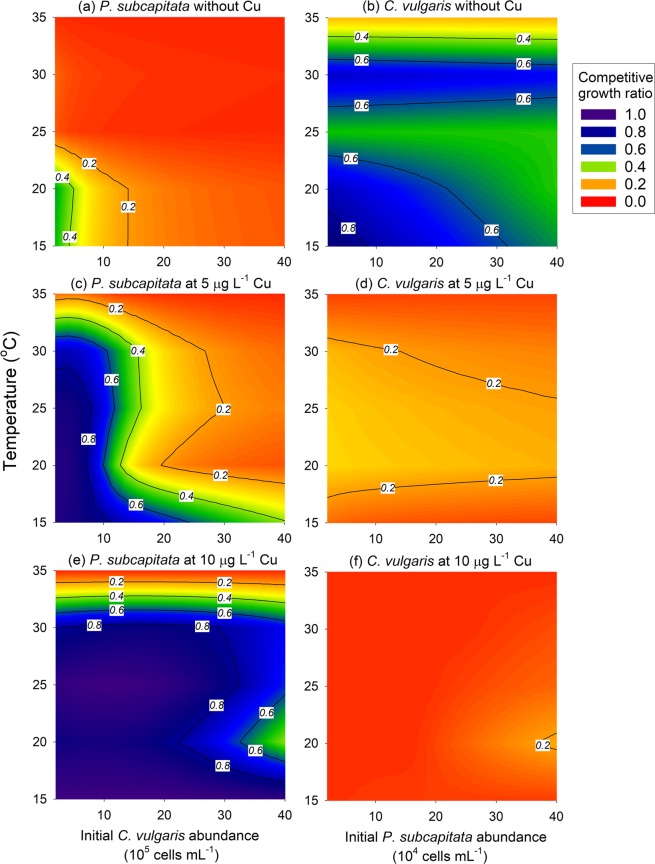
Filled contour plots of the competitive growth ratio of *Pseudokirchneriella subcapitata* and *Chlorella vulgaris*. The range of abundance of the competitor (x-axis) is plotted against temperature conditions (y-axis) in the absence of copper (panels **a**,**b**) or at 5 μg L^−1^ (**c**,**d**) or 10 μg L^−1^ copper exposure (**e**,**f**). The competitive growth rate indicates the ratio of maximum algal abundance in the coculture in comparison with the maximum abundance in single culture. These results were obtained from simulations where the initial abundance of *P. subcapitata* (**a**,**c**,**e**) and *C. vulgaris* (**b**,**d**,**f**) was 2.5 × 10^5^ and 2.5 × 10^6^ cells mL^−1^, respectively.

Changes in the initial abundance of a competitor affected the effects of temperature and copper exposure levels on the competition between *P. subcapitata* and *C. vulgaris* (Fig. 2). The predicted competitive growth ratio at each temperature and copper exposure level decreased with the increasing initial abundance of the competitor. When the initial abundance of *C. vulgaris* was <5.0 × 10^5^ cells mL^−1^, the competitive growth ratio of *P. subcapitata* was >0.41 at 15 and 20 °C in the absence of copper exposure, but this value greatly decreased as the initial abundance of *C. vulgaris* increased (Fig. 2a). The effects of the initial abundance of the competitor and temperature on the growth of *P. subcapitata* in coculture were more evident during copper exposure. At 5 μg L^−1^ copper exposure, the growth of *P. subcapitata* at 20–30 °C decelerated more strongly with increasing initial abundance of *C. vulgaris* than that at 15 °C (Fig. 2c).

The effect of the initial abundance of the competitor was noticeable only under the conditions where copper and temperature did not severely inhibit algal growth. For example, because the growth of *C. vulgaris* was greatly suppressed by copper exposure in all the tested conditions, the effects of initial abundance of the competitor and temperature were uncertain at this simulation step (Fig. 2d,f).

### Effects of initial abundance ratio and absolute initial abundance at different temperatures

**Figure 3:**
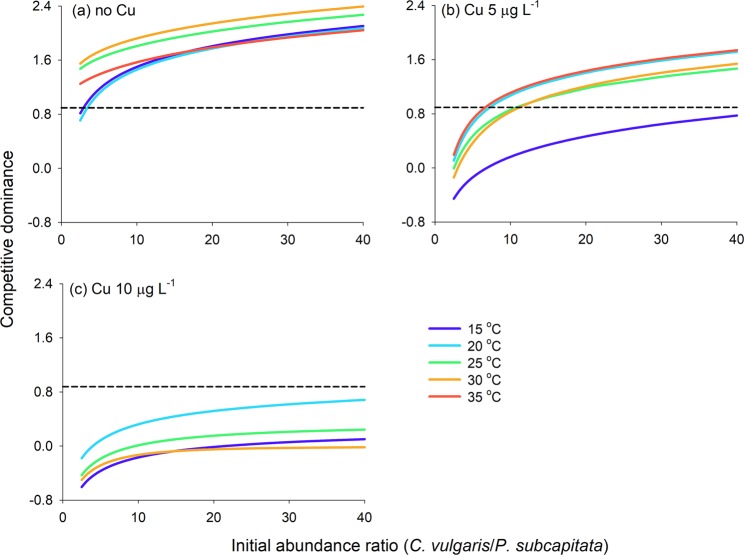
Line plots of the initial abundance ratio and competitive dominance at each temperature. The competitive dominance indicates the log_10_-transformed ratio of the maximum abundance of *Chlorella vulgaris* to that of *Pseudokirchneriella subcapitata*. The dashed line indicates the reference value of the competitive dominance, calculated from the maximum abundance of each algal species when grown individually in single culture. The results for 10 μg L^−1^ copper exposure at 35 °C were excluded because of severe inhibition of algal growth.

As the initial abundance ratio increased, the maximum abundance ratio of *C. vulgaris* and *P. subcapitata* (i.e., competitive dominance) also increased, regardless of the copper exposure levels (Fig. 3). In particular, at 5 μg L^−1^ copper, the increase in the initial abundance ratio switched the favorable species from *P. subcapitata* to *C. vulgaris* in the interference competition (Fig. 3b). At 10 μg L^−1^ copper, however, the increase in the initial abundance ratio resulted in a relatively slow increase in the competitive dominance (Fig. 3c). These results meant that owing to the inhibition of *C. vulgaris* growth by copper, the positive effect of the increased initial abundance ratio on the competitive advantage of *C. vulgaris* was limited. In contrast, the effect of temperature on the competitive dominance varied with the copper exposure levels. In the absence of copper exposure, where the conditions are favorable for *C. vulgaris* in the algal competition, the competitive dominance of *C. vulgaris* was high at 25 and 30 °C, whereas under the copper exposure conditions, it was high at 20 and 35 °C (5 μg L^−1^ copper) or 20 °C (10 μg L^−1^ copper).

**Figure 4:**
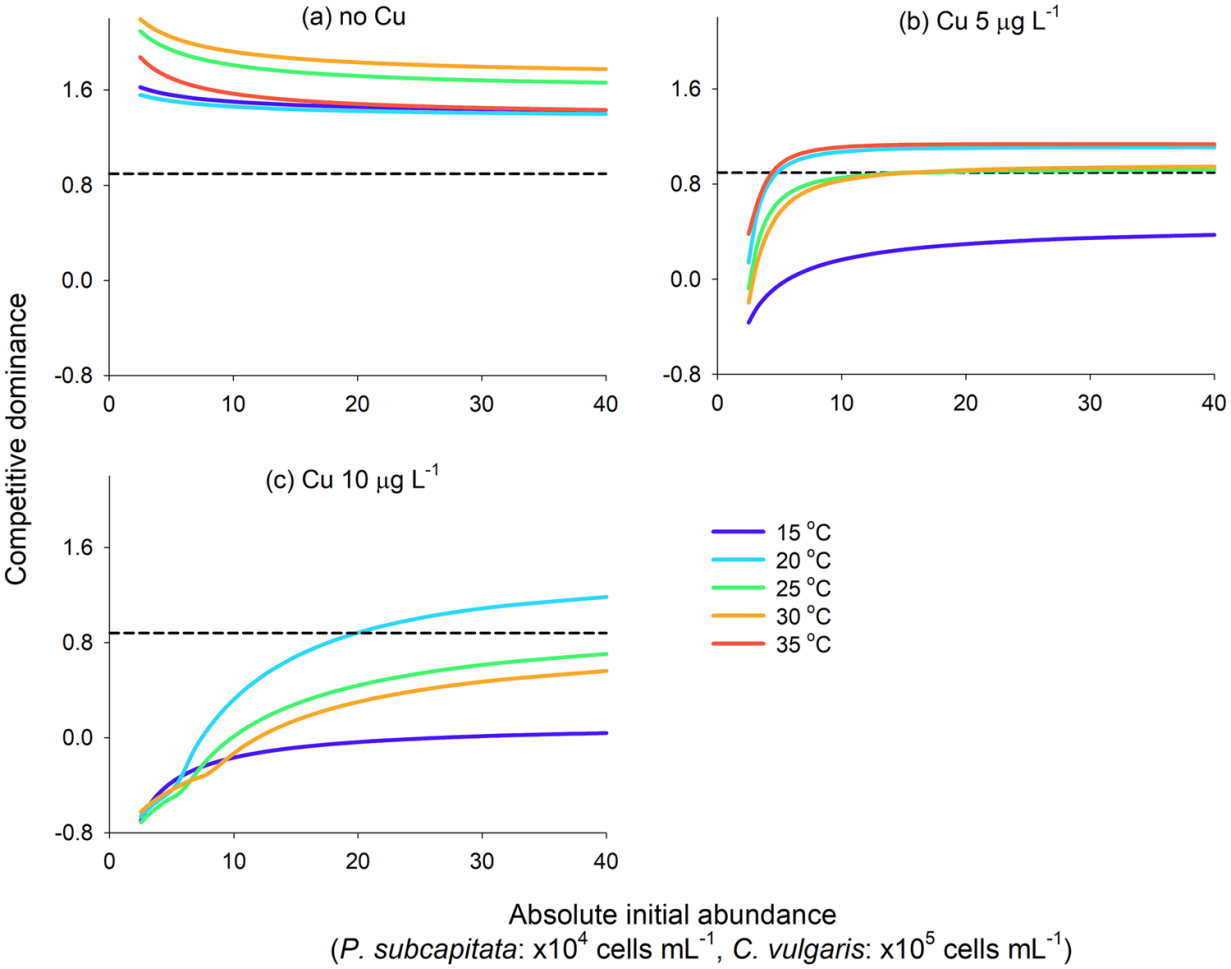
Line plots of absolute initial abundance and competitive dominance at each temperature. The competitive dominance indicates the log_10_-transformed ratio of the maximum abundance of *Chlorella vulgaris* to that of *Pseudokirchneriella subcapitata*. The dashed line indicates the reference value of the competitive dominance, calculated from the maximum abundance of each algal species when grown individually in single culture. The results for 10 μg L^−1^ copper exposure at 35 °C were excluded because of severe inhibition of algal growth.

Unlike the initial abundance ratio, the effect of the increase in absolute initial abundance on the competitive dominance varied depending on the copper exposure levels (Fig. 4). As absolute initial abundance increased, the competitive dominance of *C. vulgaris* over *P. subcapitata* decreased in the absence of copper exposure but increased during this exposure. In particular, the competitive dominance of *C. vulgaris* over *P. subcapitata* significantly increased with increasing absolute initial abundance at 20, 25, and 30 °C under 10 μg L^−1^ copper exposure (Fig. 4c). Simulation results also revealed that *C. vulgaris* could be competitive during copper exposure at temperatures above 20 °C, as the absolute initial abundance increased.

### Comparison of the effects of initial abundance levels on the competition

**Figure 5:**
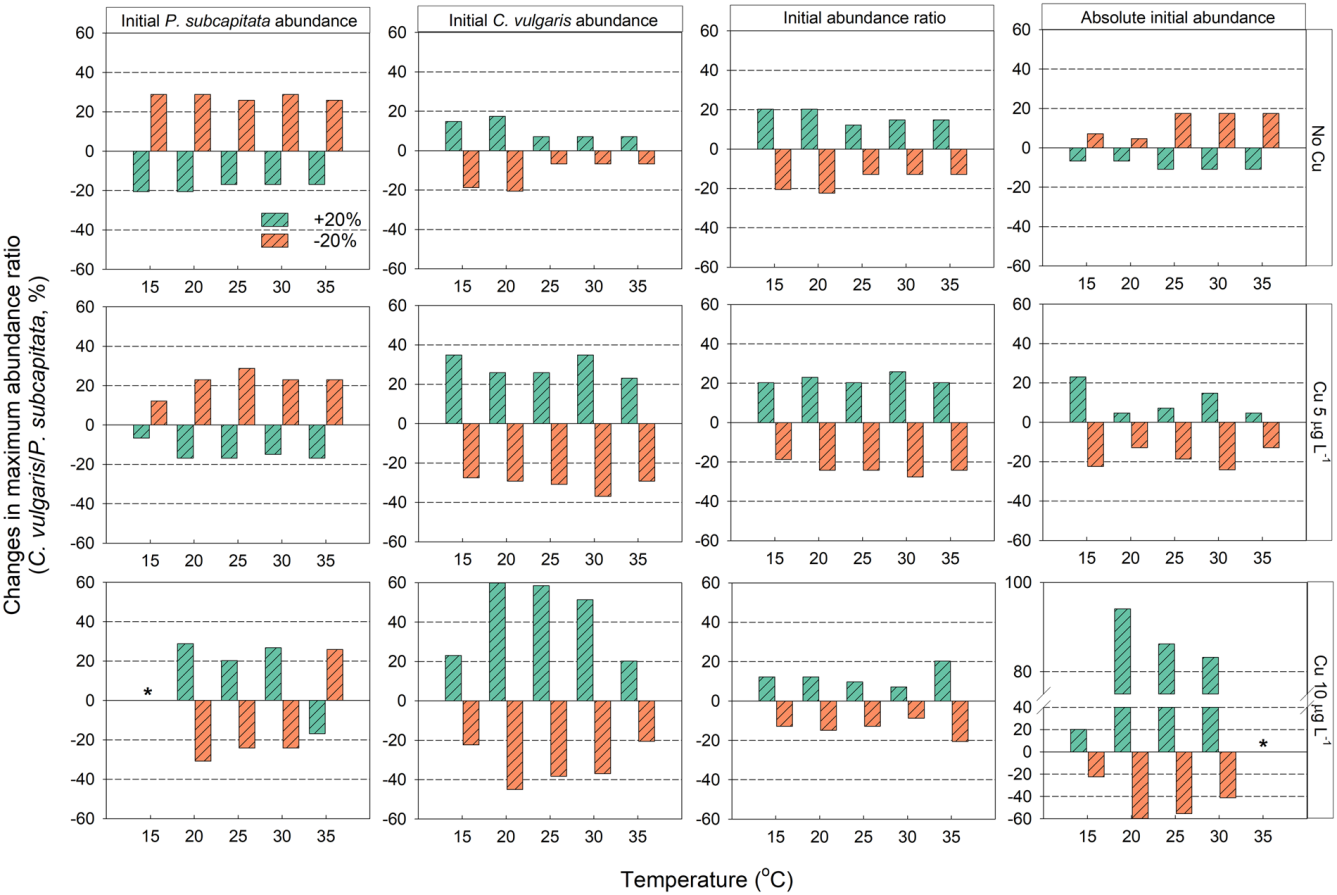
Bar plots for a comparison of the effects of an initial abundance of *Pseudokirchneriella subcapitata* and *Chlorella vulgaris*, the initial abundance ratio, and absolute initial abundance. The green bar and orange bar represent the rate of change of the maximum abundance ratio when each type of initial abundance increases and decreases by 20%, respectively. The initial abundance levels of *P. subcapitata* and *C. vulgaris* were 2.5 × 10^5^ and 2.5 × 10^6^ cells mL^−1^, respectively. Asterisks indicate that a data point was excluded from the outcome determination owing to severe inhibition of algal growth.

A comparison of the effects of initial abundance on the maximum abundance ratio at different temperatures and copper exposures is depicted in Fig. 5. The increase in the initial abundance of *P. subcapitata* had a beneficial influence on the competitive dominance of *P. subcapitata* in the absence of or at 5 μg L^−1^ copper, but it had an adverse effect during 10 μg L^−1^ copper exposure at 20–30 °C. In contrast, the beneficial effect of increased initial abundance of *C. vulgaris* on its competitive dominance was greater as the copper exposure level was raised, especially at 20–30 °C. The increase in the absolute initial abundance had a favorable effect on the competitive dominance of *P. subcapitata* and *C. vulgaris* in the absence and presence of copper exposure, respectively. In particular, the absolute initial abundance had a strong effect on the outcome of the competition between *P. subcapitata* and *C. vulgaris* at 20–30 °C, most notably at 20 °C.

## Discussion

Elevated temperatures are known to be beneficial to the competitive advantage of allelopathic algae competing with non-allelopathic algae, but our results indicate that this is true only under specific conditions. According to the simulation results based on our experimental data, trace amounts of copper entering the freshwater ecosystem can change the effects of the temperature increase on the outcome of algal competition between allelopathic and non-allelopathic species. For instance, relatively high temperatures (25 and 30 °C) were favorable for the dominance of the allelopathic alga in the absence of copper exposure, but high temperatures were rather unfavorable during 10 μg L^−1^ copper exposure. Furthermore, the initial abundance levels of the allelopathic and non-allelopathic algae exerted different effects on the algal competition depending on the temperature and copper exposure levels. This study indicates that the effect of increased temperature on the dominance of an allelopathic algal species in a freshwater ecosystem can vary with the temporal dynamics of algal abundance and various chemicals, including pollutants in aquatic ecosystems.

### Effects of temperature on the competition

Differences in minimum, maximum, or optimal temperatures among species lead to interspecific variation of biological performance, such as metabolic rate and growth, at increased temperatures^8^. Because *C. vulgaris*, an allelopathic species, is known to have a higher optimal temperature than *P. subcapitata* (*C. vulgaris*: 25.4 ± 7.0 °C and *P. subcapitata*: 23.7 ± 7.0 °C)^29^, temperature increases are expected to enhance the competitive advantage of *C. vulgaris* over *P. subcapitata*. This study clearly shows that as the temperature increases from 20 to 30 °C, the ratio of the maximum abundance of *C. vulgaris* to *P. subcapitata* increases in the absence of copper exposure (Figs. 3a and 4a). These results are consistent with previous findings that elevated temperature may promote the dominance of allelopathic species, such as *Microcystis aeruginosa*^5^, *Ostreopsis ovata*^6^, and *Leptolyngbya foveolarum*^30^, in aquatic ecosystems. By contrast, in our study, owing to the inhibition of algal growth, the competitive advantage of the allelopathic alga decreased at 35 °C, which is outside the optimal growth condition ranges of the two algal species (Supplementary Fig. S1).

Several experimental results, including the findings of this study, suggest that increased temperature is beneficial to the tested allelopathic algae rather than to their competitors in the algal competition^5,6,30^. The precise mechanism or mechanisms that account for this phenomenon are not well understood, although several possible contributing factors have been proposed. The higher optimal temperature of the allelopathic species has been named as a possible explanation in the case of cyanobacteria^31^, but Lürling *et al*.^32^ suggest that the optimal temperature ranges and growth rate of allelopathic algae at the optimum found in 67 literature sources are not significantly higher than those of the competitors. Given that temperature and other factors affect the growth of each competing alga in coculture, there is a limit to elucidation of the outcome of algal competition by means of the optimal temperature alone. For example, the growth of an algal species in coculture may be affected by the shading effect of its competitors^33^ and by changes in nutrient conditions^34^. The changes in allelochemical toxicity with temperature also make it difficult to identify the influence of temperature on the growth rate of each algal species in coculture because its trend may differ from that of the growth rate^6^. A higher optimal temperature can be considered one of the factors that enhance the competitive advantage of allelopathic algae at higher temperatures, but further studies are needed to clearly identify the key drivers of algal competition at higher temperatures.

### Effects of copper and the combined influence with effects of temperature

Of note, the increase in temperature (from 20 to 30 °C) under copper exposure reduced the competitive advantage of *C. vulgaris* as opposed to the absence of copper exposure (Figs. 3 and 4). Several factors, such as the species strain^5^ and illumination conditions^35^, have been shown to slightly modify the effect of temperature changes on the competition. Nevertheless, the factors that completely reverse the effects of temperature are not well known. The reduction in the competitive advantage of *C. vulgaris* at higher temperatures is likely associated with an increase in the toxic effects of copper at higher temperatures. Qian *et al*.^36^ have reported that an increase in the metabolic rate of algae owing to increased temperature may result in an increase in the uptake of pollutants, thereby enhancing the toxicity. Leung *et al*.^37^ have also experimentally confirmed that the effect of copper toxicity on the growth rate and photosynthetic responses of algae is strongly synergistic with increasing temperatures. Consequently, if allelopathic species are more sensitive to pollutants than their competitors, then the allelopathic algae may be excluded from the algal community at increased temperatures. In contrast, studies on community level indices, such as species composition, species richness, and Shannon diversity, have uncovered antagonistic effects of temperature increase and copper toxicity^38,39^. In addition, because the interactive effects of temperature and pollutants may vary among algal species^40^ and pollutants^41^, further research on a combination of various allelopathic algal species and pollutants is needed to understand the interaction effects on the algal competition during temperature changes.

Although differences in the sensitivity to copper between *P. subcapitata* and *C. vulgaris* were not large in this study, copper exposure had a strong effect on the outcomes of the interference competition between the two algal species. Some studies have shown that the influence of pollutants on aquatic species is greater when they are in interspecific competition^18,42,43^. According to these data, the effects of pollutants on populations may last longer during interspecific competition^43^, and subsequently, the population recovery is delayed^42^. Additionally, a reduction in resource intake owing to the competition may also increase the sensitivity of organisms to pollutants^18^. These findings support the significant difference in the competitive advantage between *P. subcapitata* and *C. vulgaris* under the copper exposure regimens that were examined in this study. Furthermore, the fact that the bioavailability and toxicity of pollutants increase with a decrease in the abundance of algae also implies that competition-induced growth inhibition can make algae vulnerable to pollutants^44^. Considering that copper can be naturally present in freshwater at concentrations ranging from 0.2 to 30 μg L^−1^, a reversal of the effects of increased temperature on the competition between allelopathic and non-allelopathic algae is likely to occur frequently in natural ecosystems^17^.

### Role of initial abundance levels of algae in the competition

Changes in the initial abundance of either the allelopathic or non-allelopathic alga produced more complicated effects of temperature and copper exposure on the two algal species (Fig. 5). Consistent with the results of another study, our results showed that the increased initial abundance of either *P. subcapitata* or *C. vulgaris* beneficially influenced their dominance^45^, but this phenomenon took place only in the absence of copper exposure. The increase in the initial abundance of both the allelopathic and non-allelopathic algae was favorable for the dominance of the allelopathic alga when the influence of copper exposure was strong (10 μg L^−1^). There are two possible reasons why the increased initial abundance levels of both algae beneficially influenced the dominance of the allelopathic alga under copper exposure. Firstly, the increased initial abundance may lead to a reduction in copper toxicity. According to Franklin *et al*.^44^, the toxicity of copper decreases as much as eight-fold when the initial abundance of *P. subcapitata* and *C. vulgaris* changes from 10^2^ to 10^5^ cells mL^−1^ because of a reduction in the amount of adsorbed copper per algal cell. Metal-binding exudates released by algal cells also reduce toxicity when algal abundance increases^44,46^. Besides, given that the characteristics of *C. vulgaris* colonies tend to protect healthy cells in the colony core, the increase in the initial abundance may enable more cells to sustain cell division^47^. Secondly, increasing the initial abundance may have enhanced the allelopathic effect in the competition. It is well known that allelopathic effects are strongly dependent on the abundance of allelopathic species^23^. The greater opportunities for contact between allelopathic and non-allelopathic algae may be another factor enhancing the allelopathy^23,48^. These effects of the initial abundance on algal competition under copper exposure are all associated with numerical abundance (i.e., algal density). For this reason, in the environment where the influence of copper was strong, the absolute initial abundance could be a more influential factor toward the outcome of the competition than the initial abundance ratio was (Figs. 3–5), as stated by Felpeto *et al*.^23^.

The favorable action of the increase in the initial abundance on the allelopathic alga at 10 μg L^−1^ copper exposure was greater at 20 °C than at 25–30 °C (Fig. 5), owing to the synergetic influence of copper with increased temperatures (subsection 4.2). This result suggests that in aquatic ecosystems exposed to pollutants such as copper, the priority effects of allelopathic algae can be rather weakened by increased temperature.

### Limitations and conclusion

This study shows for the first time through laboratory level experiments and simulations that effects of an increase in temperature on the competitive advantage of allelopathic or non-allelopathic alga can be altered by pollutants and initial abundance levels of the alga, but there are some limitations. Firstly, the model employed in this study has sufficient predictability in terms of the effect of initial algal abundance on the competition between two algal species but not in terms of copper and temperature. In other words, it is impossible to predict the outcome of competition under environmental conditions other than the copper concentration and temperature conditions under which our experimental data were obtained. The cause of this limitation is that the influence of copper and temperature on the allelochemical (chlorellin), which is a key factor in the competition between the two algal species, has not been clearly identified. Although the negative effects of allelochemicals on competitors are known^23^, definite mechanisms underlying the formation of allelochemicals and their behavior in water systems are still poorly studied^58^. Characteristics of allelochemicals, their behavior over time, and their changes with environmental conditions are the main research areas that need to be investigated in the future to more precisely model the competition between allelopathic and non-allelopathic algae. Recent advances in analytical techniques will help us to explore these areas^59^ and to develop models with better predictability. Secondly, there is a limit on the interpretation of the results of this study, which deals with a general phenomenon in a natural environment. This is because natural enemies^2^ and the complexity of communities^49^, as well as other environmental factors of an actual ecosystem, can have a significant effect on the competition among algae. Nutrient deficiencies and dynamics of nutrient concentrations, which are not considered in this study but can occur in real ecosystems, can also significantly modify the outcome of the algal competition. The limitations of extrapolating the results of the specific case to natural ecosystems should not be overlooked, but a suggestion can be derived from our findings. To precisely understand the effects of increased temperature on algal competition in a natural ecosystem, the effect of pollutants on the algal competition should not be underestimated. The fact that the difference in sensitivity between algal species varies greatly depending on the type of pollutant^50^ means that the structure of an algal community, including allelopathic algae, can be significantly affected by inflowing pollutants. The pollutants will continue to flow into aquatic ecosystems from various sources, such as industrial wastewater, landfills, mining activities, agriculture, and natural geogenic releases in the future^51^, and it may be impossible to prevent them completely. Additionally, considering that the release, fate, and exposure of pollutants may be also altered by future environmental changes^19^, clearly identifying the effects of pollutants will be essential for elucidating the algal competition in natural ecosystems.

We demonstrated that (1) increase in temperatures from 20 to 30 °C enhance the competitive advantage of the allelopathic alga only in the absence of copper exposure, (2) the influence of the increased temperatures on the competition can strongly depend on the initial abundance of two algal species and on the copper exposure level, and (3) the winner in the competition between the allelopathic and non-allelopathic algae can be switched by the combined influence of the tested factors. Accordingly, the results of our study do not support the hypothesis that increased temperatures enhance the competitive advantage of allelopathic algae in natural ecosystems. Instead, our results suggest that an increase in temperature will be beneficial to the competitive advantage of allelopathic algae when they are less susceptible to pollutants or when the algal abundance is sufficiently high. Further research on the response of allelopathic and non-allelopathic algae to various environmental factors will greatly advance the understanding of the processes promoting the dominance of allelopathic algae in an algal community during future environmental changes.

## Methods

### Tested algae and culture conditions

Two freshwater microalgal species, *P. subcapitata* (strain CCAP 278/4) and *C. vulgaris* (strain AG40003), were acquired from the Culture Collection of Algae and Protozoa (CCAP, Scottish Marine Institute, UK) and the Korean Collection for Type Cultures (Korea Research Institute of Bioscience and Biotechnology, Korea), respectively. Both algal species were maintained separately in a 200 mL Erlenmeyer flask containing 100 mL of an algal growth medium (Supplementary Table S4), prepared according to United States Environment Protection Agency method^52^ 1003.0. The flasks were incubated in a static-temperature incubator at 20 ± 1 °C, for 16 h light/8 h dark photoperiod, and at 70 μmol photons m^−2^ s^−1^ using a cool white fluorescent light.

### Experimental design

The three factors—copper exposure (three concentrations), initial abundance of the two algal species (three combinations), and temperature (five levels)—were tested to determine their effects on the competition between *P. subcapitata* and *C. vulgaris*. To measure the abundance of both algae over time in all experimental combinations, a full factorial experiment was designed. Thus, the experiments were conducted for 55 days with 45 combinations of the three factors until the growth and decline of the algal population were over. Nutrient and light conditions of the experiments were identical to the culture conditions and were not considered limiting factors during the experiments.

The exposure concentrations of copper were chosen to be lower than EC_50_ (effective concentration giving a 50% reduction in the algal growth rate relative to the control) to ensure that copper does not severely inhibit the growth of the two algal species. According to Franklin *et al*.^44^, 72 h EC_50_ values of *P. subcapitata* and *C. vulgaris* at an initial abundance of 10^5^ cells mL^−1^ at 27 °C are 17 and 16 μg L^−1^, respectively. Considering that copper concentrations below EC_50_ had a significant effect on algal competition in our previous study^22^, copper concentrations 5 and 10 μg L^−1^ were chosen as the exposure levels for the present experiments. To prepare the copper-containing test media, a copper stock solution was made up by dissolving reagent grade copper (II) sulfate pentahydrate (CuSO_4_·5H_2_O, ≥99% purity, Sigma-Aldrich) in the algal growth medium, and then, the mixture was diluted with the medium to obtain the final exposure concentrations of 0 (control), 5, and 10 μg L^−1^. The pH of the test medium was adjusted to 7.5 ± 0.1 with 0.1 M HCl or NaOH.

The algal growth experiments were carried out in a 100 mL glass beaker filled with 70 mL of the test medium along with three combinations of the initial abundance levels of the algae. The initial abundance levels of *P. subcapitata* and *C. vulgaris* in the three combinations were set up as follows: 2.5% and 10% (Combination A), 5% and 5% (Combination B), and 10% and 2.5% (Combination C) of the maximum abundance levels of each species in single culture (*P. subcapitata*: 5.0 × 10^6^ cells mL^−1^, and *C. vulgaris*: 4.0 × 10^7^ cells mL^−1^). To inoculate an algal amount corresponding to the determined initial abundance, *P. subcapitata* and *C. vulgaris* were sampled from steady-state algal cultures in which abundance was measured. The test beakers were sealed with parafilm (polyethylene) to prevent evaporation of the test solution and were placed separately in static-temperature incubators maintained at 15, 20, 25, 30, or 35 °C. To minimize the sedimentation and flocculation of the algal cells, the test beakers were manually agitated twice a day with the cover removed in the incubator. Five biological replicates were used for each of the 45 experimental conditions (*n* = 5).

During the 55 days of the experiment, the abundance of *P. subcapitata* and *C. vulgaris* was checked periodically (16 times total; until 3 weeks of exposure: every 1 to 4 days, thereafter: every 5 to 8 days). The abundance of algal cells in each beaker was measured five times by counting the algal cells in a sampled 5 μL algal suspension, using a hemocytometer (Marienfeld, Germany) under an optical microscope (E200; Nikon, Japan). Given that the two algal species have different shapes and sizes, the abundance of each species could be counted separately in a mixture under the microscope^22^.

It should be noted that no statistical analysis was performed to evaluate the effects of the tested factors on algal growth because the purpose of the experiments was to obtain experimental data for estimating the parameters of the model to be used in the simulations. Therefore, instead of hypothesis testing by means of the observed data, an evaluation of model performance was performed as described below.

### Mathematical model and parameter estimation

To assess the influence of the copper exposure level, initial algal abundance, and temperature on the competition between the two algal species, the mathematical model previously developed by Kim *et al*.^22^ was utilized for the simulations. The model was employed to describe the dynamics of abundance levels of *P. subcapitata* and *C. vulgaris* during interference competition, including the allelopathic effect of *C. vulgaris*, which inhibits the growth of *P. subcapitata* by producing an allelochemical called chlorellin^53^. The finding that chlorellin not only strongly inhibits the growth of *P. subcapitata* but also weakly inhibits the growth of *C. vulgaris* itself was taken into account in the model^26^. Because the abundance levels of *P. subcapitata* and *C. vulgaris* could not reach an equilibrium owing to the strong interference competition between the two algal species via chlorellin^25^, the model was designed to simulate transient dynamics of the algal abundance. The model is based on three ordinary differential equations representing algal abundance (*X*)^22,25^, allelochemical concentration (*p*)^54^, and habitat quality (*S*)^22^, as follows:

1 $${\dot{X}}_{i}=-\,{d}_{i}(T){X}_{i}+{X}_{i}\left\{\begin{array}{cc}{\mu }_{i}(T)\exp (-{r}_{i}p)\left(1-\frac{{k}_{i}(T)}{{c}_{i}{X}_{i,0}+{c}_{j}{X}_{j,0}}\right)S & {\rm{for}}\,S > 0\\ -{a}_{i}(T)\frac{{X}_{i,{\rm{m}}}-{X}_{i}}{{X}_{i,{\rm{m}}}} & \,{\rm{otherwise}}.\end{array}\right\},$$

2 $$\dot{p}=\{\begin{array}{cc}\alpha (T){\dot{X}}_{{\rm{cv}}} & {\rm{for}}\,{\dot{X}}_{{\rm{cv}}} > 0\\ 0 & {\rm{otherwise}}.\end{array}\},$$

3 $$\dot{S}=-\,{m}_{i}{X}_{i}-{m}_{j}{X}_{j}-\{\begin{array}{cc}{\dot{X}}_{i}{n}_{i}+{\dot{X}}_{j}{n}_{j} & {\rm{for}}\,{\dot{X}}_{i} > 0\,{\rm{and}}\,{\dot{X}}_{j} > 0\\ 0 & {\rm{otherwise}}.\end{array}\},$$

where the subscripts denote the following: a particular algal species, *i*; competitor species, *j*; initial abundance, 0; maximum abundance under the given conditions, m; and *C. vulgaris*, cv, which is a chlorellin-producing species. In Eq. (1), the changes in algal abundance, *Ẋ*_*i*_, are determined by population growth, reflecting the temperature-dependent growth rate, μ_*i*_(*T*), growth inhibition by chlorellin, exp(−*r*_*i*_*p*), copper toxicity, 1 − (*k*_*i*_(*T*)/(*c*_*i*_*X*_*i*,0_ + *c*_*j*_*X*_*j*,0_)), and habitat quality, *S*, as well as by population decline via temperature-dependent mortality, *d*_*i*_(*T*). Because chlorellin has a much greater effect on the growth of *P. subcapitata* than *C. vulgaris*^26^, the growth inhibition constant (*r*_*i*_) is greater for *P. subcapitata* than for *C. vulgaris*. When the habitat quality reaches zero, the algal abundance decreases rapidly as population collapse, i.e., *a*_*i*_(*T*)((*X*_*i*,m_ − *X*_*i*_)/*X*_*i*,m_), is added to the mortality. In Eq. (2), the concentration of chlorellin, *p*, has a linear relationship with the growth of *C. vulgaris*, *Ẋ*_cv_, and *α*(*T*) as a temperature-dependent coefficient. In Eq. (3), habitat quality *S* starts from 1 and decreases gradually with the degradation of the habitat because of the depletion of nutrients and accumulation of toxic waste during the growth (*Ẋ*_*i*_*n*_*i*_ and *Ẋ*_*j*_*n*_*j*_) and metabolism (*m*_*i*_*X*_*i*_ and *m*_*j*_*X*_*j*_) of the two algal species. *S* is conceptual representation of habitat quality of the coculture, is estimated by the observed data, and does not mean the actual concentration of nutrients or waste. Detailed descriptions of the parameters and units are given in Supplementary Table S5. More detailed descriptions of the model development and assumptions can be found in our previous study^22^.

The models described above were simulated using the Powersim studio (Powersim software AS, Norway) by the Euler integration method with 0.01 time-step in days. To calibrate the model using the observed data, the values of the parameters minimizing the sum of square error (the difference between the observed and simulated algal abundance) were estimated via the Powersim solver analysis tools. Five temperature-dependent parameters, i.e., the growth rate, μ_*i*_(*T*), mortality, *d*_*i*_(*T*), the abundance decrease rate, *a*_*i*_(*T*), the chlorellin formation coefficient, *α*(*T*), and the copper toxicity coefficient, *k*_*i*_(*T*), were estimated individually for each temperature condition. Other temperature-independent parameters, such as the habitat depletion rate (*m*_*i*_ and *n*_*i*_), the inhibition constant (*r*_*i*_), and the contribution rate toward copper bioavailability (*c*_*i*_), were assumed (estimation) to have the same values at all temperatures.

The performance of the calibrated model was evaluated in three ways by means of PBIAS, the IoA, and ME^27,28^. PBIAS measures the average tendency of the predicted data to be greater or less than the observed data (Eq. (4)). The optimal value of PBIAS is 0%, with a lower value indicating a more accurate prediction. The IoA (Eq. (5)) and ME (Eq. (6)) take a value between 0 and 1, and a higher value indicates that the model predictions better describe the observed data.

4 $${\rm{PBIAS}}=\frac{{\sum }_{i=1}^{n}({P}_{i}-{O}_{i})}{{\sum }_{i=1}^{n}{O}_{i}}\times 100 \% $$

5 $${\rm{IoA}}=1-\frac{{\sum }_{i=1}^{n}{({O}_{i}-{P}_{i})}^{2}}{{\sum }_{i=1}^{n}{(|{P}_{i}-\bar{O}|+|{O}_{i}-\bar{O}|)}^{2}}$$

6 $${\rm{ME}}=1-\frac{{\sum }_{i=1}^{n}{({O}_{i}-{P}_{i})}^{2}}{{\sum }_{i=1}^{n}{({O}_{i}-\bar{O})}^{2}},$$

where *n* is the number of observed values, *O*_*i*_ is the *i*^th^ observed value, *P*_*i*_ is the *i*^th^ predicted value, and *Ō* is the mean of the observed values.

### Simulations

After the models were calibrated, a simulation analysis was performed to explore the effects of temperature, initial algal abundance, and copper exposure levels on interference competition between *P. subcapitata* (the non-allelopathic alga) and *C. vulgaris* (the allelopathic alga). To quantitatively describe the effect of each condition on the competition, two indices—competitive growth ratio and competitive dominance—were calculated from the predicted algal abundance levels. The competitive growth ratio is an indicator of how much the algal growth has been inhibited by the competitor and enables a sensible comparison when the difference in the carrying capacity between two species is substantial, as in the case of *P. subcapitata* and *C. vulgaris*^55^. This index was calculated as the ratio of simulated abundance in the coculture to the optimal maximum abundance in the single culture that was already known (*P. subcapitata*: 5.0 × 10^6^ cells mL^−1^, and *C. vulgaris*: 4.0 × 10^7^ cells mL^−1^). The competitive dominance denotes the ratio of maximum abundance of each species in the coculture. This index, unlike the competitive growth ratio, does not indicate inhibition of the growth of allelopathic or non-allelopathic algae owing to the competition but shows which alga has an advantage under given conditions. The competitive dominance was calculated as the log_10_-transformed ratio of the maximum abundance of *C. vulgaris* to that of *P. subcapitata*. To effectively identify the inhibition of algal growth by the interference competition, the log-transformed value of the ratio of the observed maximum abundance of *P. subcapitata* and *C. vulgaris* in the single culture was designated as a reference value.

Initial abundance is a broad concept that involves ratios and absolute values of one or more species. To thoroughly investigate the influence of the initial abundance in the simulation analysis, the initial abundance was classified as the initial abundance of the competitor, the initial abundance ratio of *P. subcapitata* and *C. vulgaris*, and absolute initial abundance (the sum of abundance levels of the two algal species). Their effects on the competition were sequentially analyzed through the simulation of the abundance of *P. subcapitata* and *C. vulgaris* in coculture in each initial abundance condition. First, to compare the effects of different temperatures on the competition between *P. subcapitata* and *C. vulgaris*, changes in abundance of the two algal species in the coculture were simulated at fixed initial abundance of both species at each copper exposure level and temperature. In this simulation, the initial abundance levels of *P. subcapitata* and *C. vulgaris* were fixed at 2.5 × 10^5^ and 2.5 × 10^6^ cells mL^−1^, respectively (Simulation I in Supplementary Fig. S4), i.e., ~5% of their maximum abundance levels, and the abundance levels of each species were simulated for 55 d, the same experimental period at each temperature and copper exposure level. Second, a simulation was performed to assess the effect of changes in the initial abundance of their competitor on the competition outcome. In this simulation, the initial abundance of *P. subcapitata* and *C. vulgaris* was 2.5 × 10^5^ and 2.5 × 10^6^ cells mL^−1^, respectively, as in Simulation I. The initial abundance of the competitors tested ranged from 2.0 × 10^4^ to 4.0 × 10^5^ cells mL^−1^ for *P. subcapitata* and from 2.0 × 10^5^ to 4.0 × 10^6^ cells mL^−1^ for *C. vulgaris* (Simulation II in Supplementary Fig. S4). Third, the changes in the competitive dominance in the coculture were simulated via changes in the initial abundance ratio under the given environmental conditions. The initial abundance ratios of *C. vulgaris* to *P. subcapitata*, ranging from 2.5 to 40, were tested by adjustment of the initial abundance of *C. vulgaris* and *P. subcapitata* from 1.0 × 10^6^ to 4.0 × 10^6^ and from 4.0 × 10^5^ to 1.0 × 10^5^ cells mL^−1^, respectively (Simulation III in Supplementary Fig. S3). Lastly, the influence of absolute initial abundance on the competitive dominance was analyzed via adjustment of the initial *P. subcapitata* abundance from 2.5 × 10^4^ to 4.0 × 10^5^ cells mL^−1^ and that of *C. vulgaris* from 2.5 × 10^5^ to 4.0 × 10^6^ cells mL^−1^ (Simulation IV in Supplementary Fig. S4). The range of initial abundance levels of *P. subcapitata* and *C. vulgaris* covered by each simulation can be found in Supplementary Fig. S4.

To compare the effects of each type of initial abundance on the outcome of the algal competition, a local sensitivity analysis was performed. The sensitivity analysis was conducted via a disturbance method through changes in the initial value of a target variable (±20% of the default value), with calculation of the sensitivity (*S*) as follows^56,57^:

7 $$S=\frac{\partial X/X}{\partial Y/Y}$$

where *X* is the maximum abundance ratio of *C. vulgaris* to *P. subcapitata* under given conditions, and *Y* is the target variable. The target variables were initial *P. subcapitata* abundance, initial *C. vulgaris* abundance, the initial abundance ratio, and absolute initial abundance. The default values of the initial abundance of *P. subcapitata* and *C. vulgaris* were 2.5 × 10^5^ and 2.5 × 10^6^ cells mL^−1^, respectively, as in Simulation I. Sensitivity was analyzed for all combinations of temperatures and copper exposure levels.

## Supplementary information

Supplementary Information.

## Data Availability

All data generated and analyzed during this study are included in this published article (and its Supplementary Information Files) and are also available from the corresponding author on reasonable request.
