# Supplementary material for: Temperature-dependent competitive advantages of an allelopathic alga over non-allelopathic alga are altered by pollutants and initial algal abundance levels
Source: Sci Rep. 2020 Mar 10;10:4419. doi: 10.1038/s41598-020-61438-9 (PMC7064544; doi:10.1038/s41598-020-61438-9)
Supplement: Supplementary file 1 — Supplementary Information. [file 41598_2020_61438_MOESM1_ESM.pdf]

Supplementary Information

Temperature-dependent competitive advantages of an allelopathic alga over non-allelopathic alga are altered by pollutants and initial algal abundance levels

Yongeun Kim[1], Jino Son[1], Yun-Sik Lee[1], June Wee[2], Minyoung Lee[2], Kijong Cho[2,*]

[1] Ojeong Resilience Institute, Korea University, Seoul 02841, Republic of Korea

[2] Department of Environmental Science and Ecological Engineering, Korea University, Seoul 02841, Republic of Korea

Experimental data and model parameters

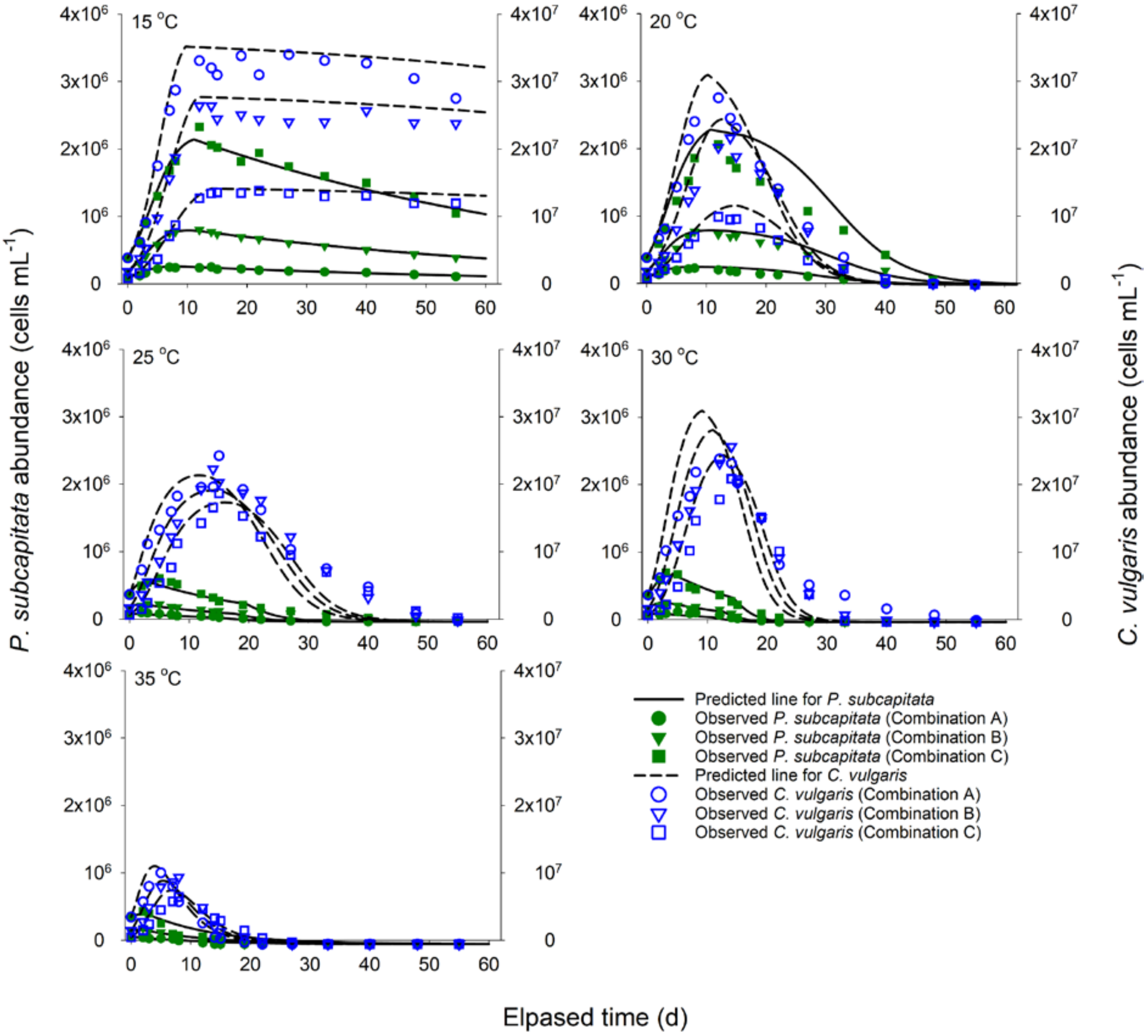

Fig. S1 Predicted and observed abundances in the absence of copper exposure with three combinations of initial abundances of *Pseudokirchneriella subcapitata* (predicted: solid line, observed: closed symbol) and *Chlorella vulgaris* (predicted: dashed line, observed: opened symbol) at various temperatures. Each symbol represents the initial abundances of *P. subcapitata* and *C. vulgaris*: circle (Combination A): 1.25×10^5 cells mL^{-1}, 4.0×10^6 cells mL^{-1}; inverted triangle (Combination B): 2.5×10^5 cells mL^{-1}, 2.0×10^6 cells mL^{-1}; square (Combination C): 5.0×10^5 cells mL^{-1}, 1.0×10^6 cells mL^{-1}, respectively.

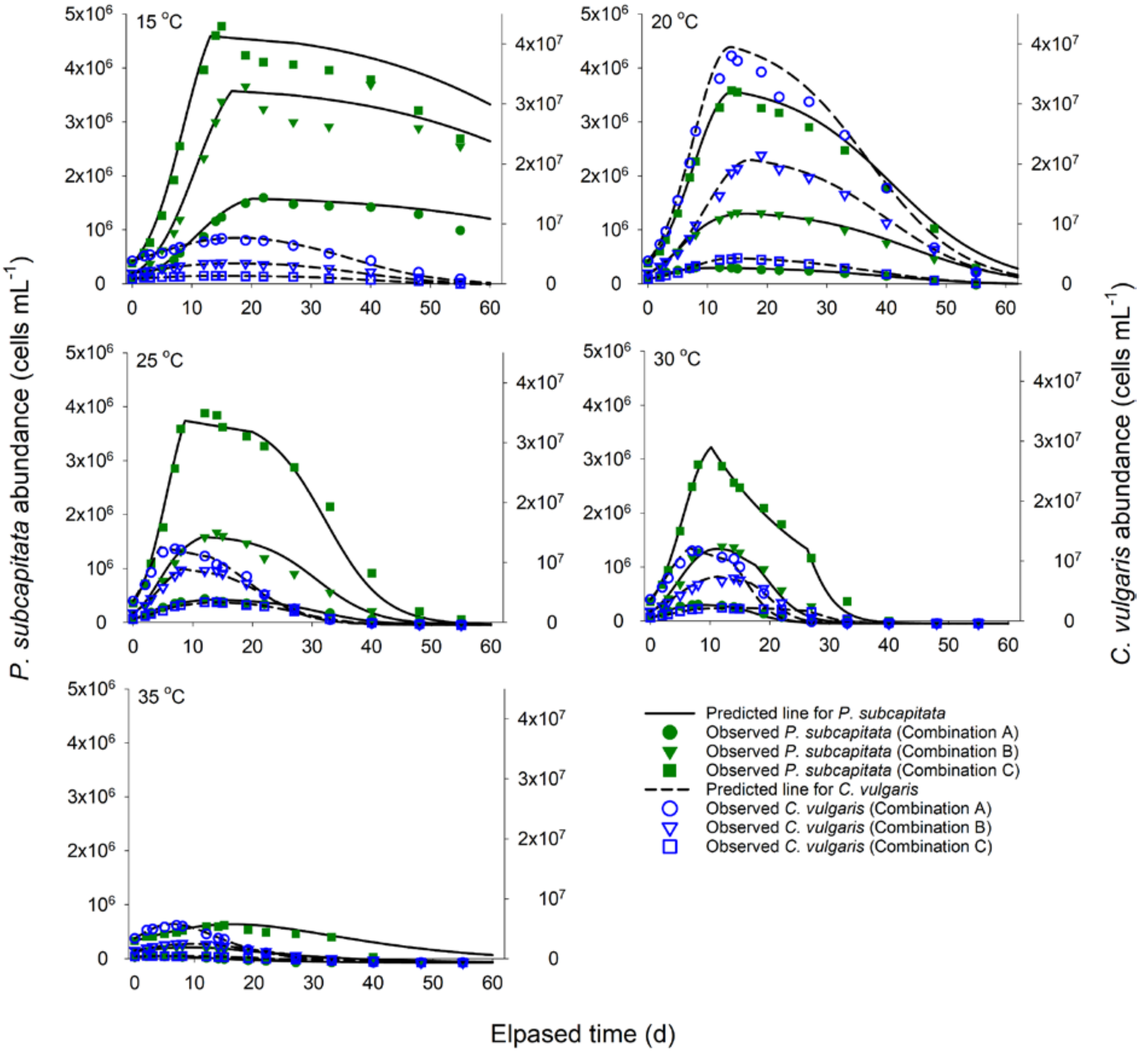

Fig. S2 Predicted and observed abundances at 5 μg L^{-1} copper-exposed conditions with three combinations of initial abundances of *Pseudokirchneriella subcapitata* (predicted: solid line, observed: closed symbol) and *Chlorella vulgaris* (predicted: dashed line, observed: opened symbol) at various temperatures. Each symbol represents the initial abundances of *P. subcapitata* and *C. vulgaris*: circle (Combination A): 1.25×10^5 cells mL^{-1}, 4.0×10^6 cells mL^{-1}; inverted triangle (Combination B): 2.5×10^5 cells mL^{-1}, 2.0×10^6 cells mL^{-1}; square (Combination C): 5.0×10^5 cells mL^{-1}, 1.0×10^6 cells mL^{-1}, respectively.

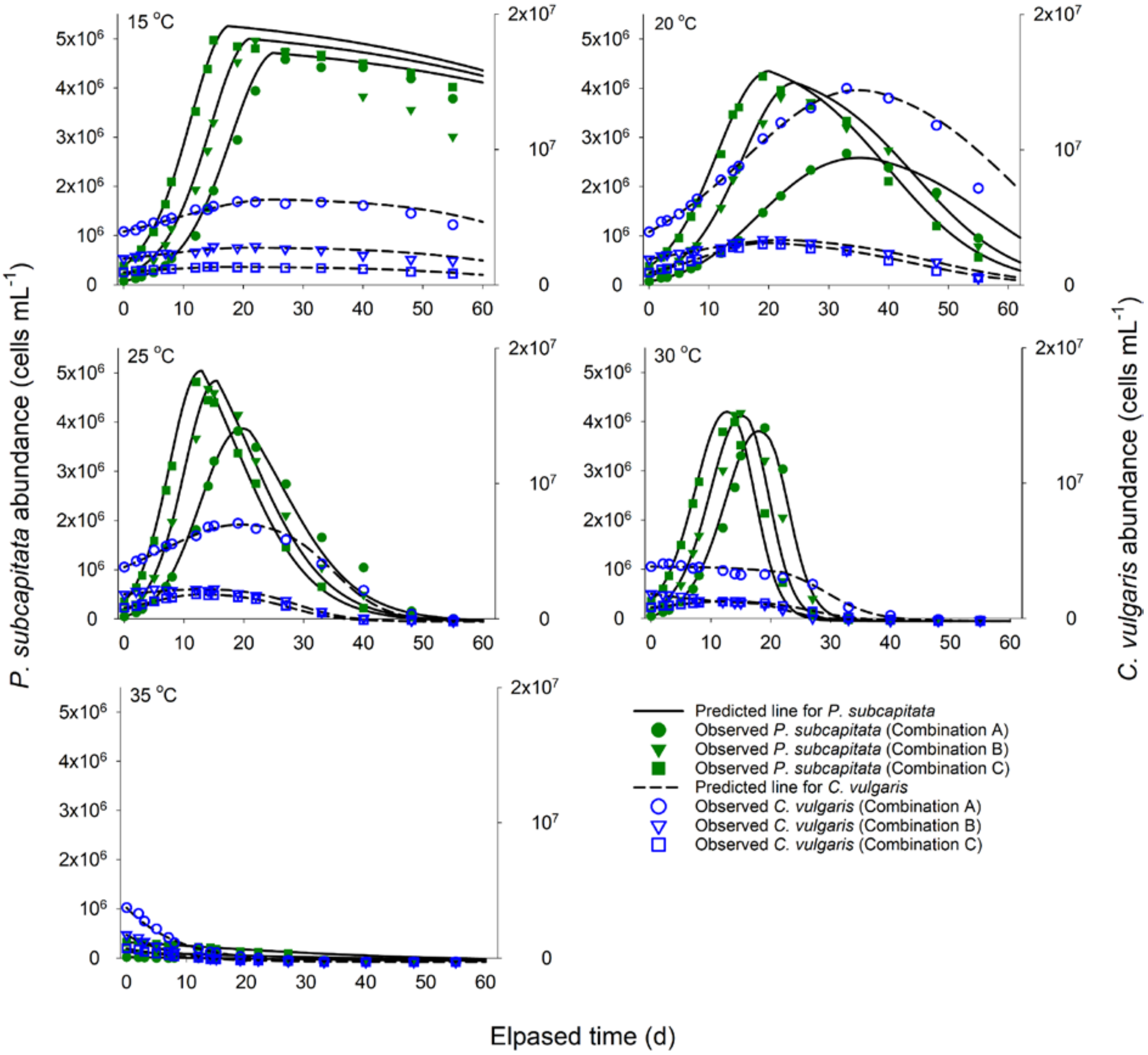

Fig. S3 Predicted and observed abundances at 10 µg mL^{-1} copper-exposed conditions with three combinations of initial abundances of *Pseudokirchneriella subcapitata* (predicted: solid line, observed: closed symbol) and *Chlorella vulgaris* (predicted: dashed line, observed: opened symbol) at various temperatures. Each symbol represents the initial abundances of *P. subcapitata* and *C. vulgaris*: circle (Combination A): 1.25 × 10^{5} cells mL^{-1}, 4.0 × 10^{6} cells mL^{-1}; inverted triangle (Combination B): 2.5 × 10^{5} cells mL^{-1}, 2.0 × 10^{6} cells mL^{-1}; square (Combination C): 5.0 × 10^{5} cells mL^{-1}, 1.0 × 10^{6} cells mL^{-1}, respectively.

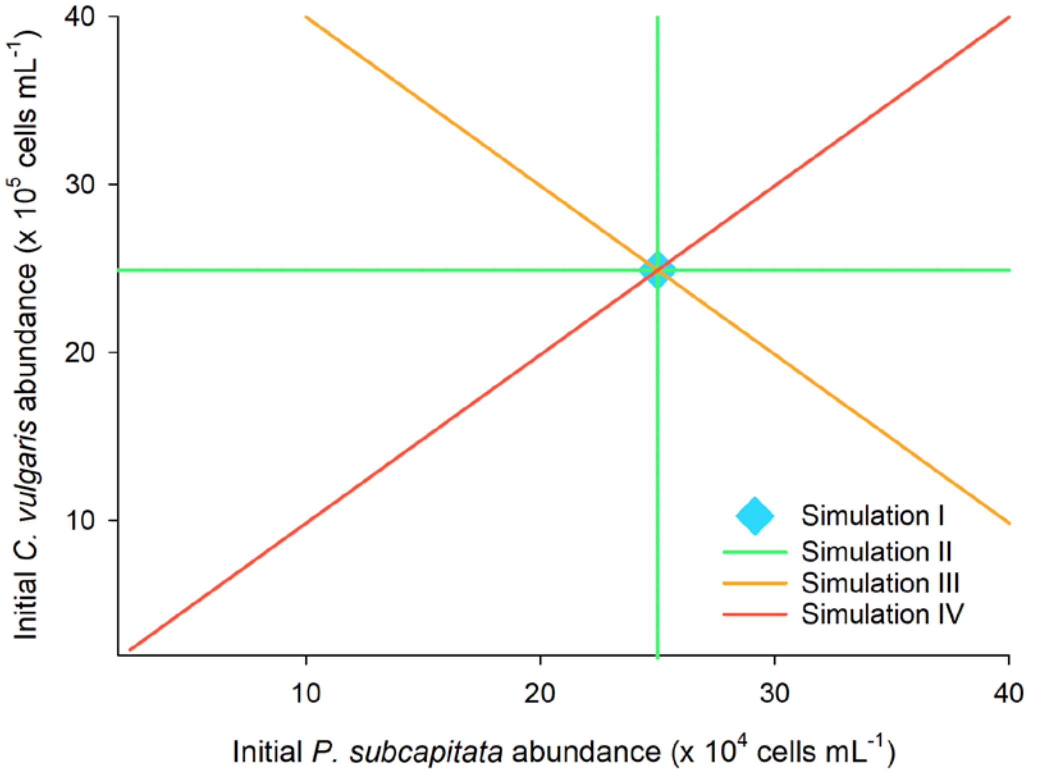

Fig. S4 Line plot showing the range of initial abundances of *Pseudokirchneriella subcapitata* and *Chlorella vulgaris* covered by each simulation study. Simulation I: Simulation with a fixed initial abundance of both algal species (the initial abundances of *P. subcapitata* and *C. vulgaris* were fixed at 2.5×10^5 cells mL^{-1} and 2.5×10^6 cells mL^{-1}, respectively), Simulation II: Simulation with changing competitor's initial abundance (the initial abundance of the competitors tested ranging from 2.0×10^4 to 4.0×10^5 cells mL^{-1} for *P. subcapitata* and 2.0×10^5 to 4.0×10^6 cells mL^{-1} for *C. vulgaris*), Simulation III: Simulation with changing the initial abundance ratios (the initial abundance ratios of *C. vulgaris* to *P. subcapitata* tested ranging from 2.5 to 40), Simulation IV: Simulation with changing the absolute initial abundance (the absolute initial abundance was tested by adjusting the initial abundance of *P. subcapitata* from 2.5×10^4 to 4.0×10^5 cells mL^{-1} and *C. vulgaris* from 2.5×10^5 to 4.0×10^6 cells mL^{-1}).

Table S1 Estimated model parameters of *Pseudokirchneriella subcapitata* for each experimental condition

		Variable parameter				Fixed parameter			
Condition	Temp. (°C)	$\mu_i(T)$ (d^{-1})	$d_i(T)$ (d^{-1})	$a_i(T)$ (d^{-1})	$k_i(T)$ (cells mL^{-1})	m_i (mL $cells^{-1}$)	n_i (mL $cells^{-1}$)	r_i (μg^{-1})	c_i
Control	15	0.36	0.01	0.01					
	20	0.39	0.01	0.16					
	25	0.41	0.05	0.23	-	7.07×10^{-11}	2.53×10^{-7}	8.98	-
	30	0.39	0.07	0.41					
	35	0.20	0.09	1.03					
Cu 5 μg L^{-1}	15	0.30	0.01	0.06	1.00				
	20	0.30	0.01	0.11	3.33				
	25	0.43	0.01	0.20	2.20	1.95×10^{-9}	6.30×10^{-8}	8.98	5.34×10^{-5}
	30	0.44	0.05	0.29	2.20				
	35	0.18	0.10	0.35	2.41				
Cu 10 μg L^{-1}	15	0.23	0.01	0.03	0.10				
	20	0.23	0.01	0.11	4.76				
	25	0.41	0.05	0.11	4.76	1.39×10^{-8}	6.23×10^{-8}	8.98	1.68×10^{-4}
	30	0.42	0.08	0.40	4.76				
	35	0.10	0.11	0.40	5.19				

Table S2 Estimated model parameters of *Chlorella vulgaris* for each experimental condition

Condition	Temp. (°C)	Variable parameter $\mu_i(T)$ (d^{-1})	$d_i(T)$ (d^{-1})	$a_i(T)$ (d^{-1})	$k_i(T)$ (cells mL^{-1})	$\alpha(T)$ (μg $cells^{-1}$)	Fixed parameter m_i (mL $cells^{-1}$)	n_i (mL $cells^{-1}$)	r_i (μg^{-1})	c_i
Control	15	0.40	0.01	0.02	-	8.66×10^{-12}	3.16×10^{-9}	8.20×10^{-9}	1.10	-
	20	0.41	0.02	0.24		1.22×10^{-11}				
	25	0.62	0.02	0.24		8.40×10^{-11}				
	30	0.62	0.02	0.40		3.36×10^{-11}				
	35	0.77	0.35	0.79		3.36×10^{-11}				
Cu 5 μg L^{-1}	15	0.10	0.01	0.12	20.24	5.72×10^{-12}	2.78×10^{-9}	5.30×10^{-10}	1.10	3.99×10^{-5}
	20	0.37	0.01	0.12	31.48	9.40×10^{-12}				
	25	0.51	0.02	0.20	32.71	4.57×10^{-12}				
	30	0.40	0.03	0.42	32.83	4.53×10^{-12}				
	35	0.37	0.14	0.53	32.83	2.64×10^{-12}				
Cu 10 μg L^{-1}	15	0.05	0.01	0.09	25.18	4.59×10^{-12}	4.73×10^{-11}	2.85×10^{-10}	1.10	1.19×10^{-5}
	20	0.26	0.01	0.10	47.71	8.41×10^{-12}				
	25	0.27	0.01	0.22	51.31	9.65×10^{-12}				
	30	0.26	0.01	0.23	61.66	8.28×10^{-12}				
	35	0.10	0.12	0.18	61.25	3.21×10^{-12}				

Table S3 Measured performances of the calibrated model for abundances of *Pseudokirchneriella subcapitata* and *Chlorella vulgaris* in co-culture. PBIAS, percent bias; IoA, index of agreement; ME, model efficiency

	P. subcapitata			*C. vulgaris*		
Condition	PBIAS (%)	IoA	ME	PBIAS (%)	IoA	ME
No Cu	7.45	0.980	0.955	6.52	0.973	0.928
Cu 5 μg L^{-1}	4.22	0.986	0.976	2.99	0.988	0.982
Cu 10 μg L^{-1}	4.60	0.986	0.974	1.31	0.988	0.982

Table S4 Concentration of macronutrients and micronutrients in the culture medium (USEPA method 1003.0)

Macronutrient	Concentration (mg L^{-1})	Micronutrient[1)]	Concentration (μg L^{-1})
$NaNO_3$	25.5	H_3BO_3	185.0
$MgCl_2·6H_2O$	12.2	$MnCl_2·4H_2O$	416.0
$CaCl_2·2H_2O$	4.41	$ZnCl_2$	3.27
MgSO4·$7H_2O$	14.7	$CoCl_2·6H_2O$	1.43
K_2HPO_4	1.04	$CuCl_2·2H_2O$	0.012
$NaHCO_3$	15.0	$Na_2MoO_4·2H_2O$	7.26
		$FeCl_3·6H_2O$	160.0
		Na_2SeO_4	2.39

[1)] To prevent copper chelation, EDTA (ethylenediaminetetraacetic acid) was omitted from the original culture medium.

Table S5 A list of the model parameter descriptions and units

Parameter[1)]	Description	Units
$\mu_i(T)$	Temperature-dependent specific growth rate	d^{-1}
r_i	Growth-inhibition constant	μg^{-1}
$k_i(T)$	Temperature-dependent copper toxicity coefficient	cells mL^{-1}
c_i, c_j	Specific contribution rate on copper bioavailability	-
$a_i(T)$	Temperature-dependent decrease rate	d^{-1}
$X_{i,0}$, $X_{j,0}$	Initial abundance of algae	cells mL^{-1}
$X_{i,\mathrm{m}}$	Maximum algal abundance under the given condition	cells mL^{-1}
$d_i(T)$	Temperature-dependent mortality	d^{-1}
m_i, m_j	Habitat depletion rate of the cell maintenance	mL $cells^{-1}$
n_i, n_j	Habitat depletion rate of the algal growth	mL $cells^{-1}$
$\alpha(T)$	Temperature-dependent chlorellin formation coefficient	μg $cells^{-1}$

[1)] Subscript '*i*' and '*j*' indicate the parameters related to the particular algae species and their competitor, respectively.
